# Overview of Metabolomic Analysis and the Integration with Multi-Omics for Economic Traits in Cattle

**DOI:** 10.3390/metabo11110753

**Published:** 2021-10-30

**Authors:** Dan Hao, Jiangsong Bai, Jianyong Du, Xiaoping Wu, Bo Thomsen, Hongding Gao, Guosheng Su, Xiao Wang

**Affiliations:** 1 Beijing Zhongnongtongchuang (ZNTC) Biotechnology Co., Ltd., Beijing 100193, China; danhao@mbg.au.dk (D.H.); baijiangsong@163.com (J.B.); jianyongdu788@sohu.com (J.D.); xpwu594419341@gmail.com (X.W.); 2 Shijiazhuang Zhongnongtongchuang (ZNTC) Biotechnology Co., Ltd., Shijiazhuang 052463, China; 3 Department of Molecular Biology and Genetics, Aarhus University, 8000 Aarhus, Denmark; bo.thomsen@mbg.au.dk; 4 College of Veterinary Medicine, China Agricultural University, Beijing 100193, China; 5 Center for Quantitative Genetics and Genomics, Aarhus University, 8830 Tjele, Denmark; hongding.gao@qgg.au.dk (H.G.); guosheng.su@qgg.au.dk (G.S.); 6 Konge Larsen ApS, 2800 Kongens Lyngby, Denmark

**Keywords:** cattle, metabolomics, multi-omics, integrated analysis, economic trait, review

## Abstract

Metabolomics has been applied to measure the dynamic metabolic responses, to understand the systematic biological networks, to reveal the potential genetic architecture, etc., for human diseases and livestock traits. For example, the current published results include the detected relevant candidate metabolites, identified metabolic pathways, potential systematic networks, etc., for different cattle traits that can be applied for further metabolomic and integrated omics studies. Therefore, summarizing the applications of metabolomics for economic traits is required in cattle. We here provide a comprehensive review about metabolomic analysis and its integration with other omics in five aspects: (1) characterization of the metabolomic profile of cattle; (2) metabolomic applications in cattle; (3) integrated metabolomic analysis with other omics; (4) methods and tools in metabolomic analysis; and (5) further potentialities. The review aims to investigate the existing metabolomic studies by highlighting the results in cattle, integrated with other omics studies, to understand the metabolic mechanisms underlying the economic traits and to provide useful information for further research and practical breeding programs in cattle.

## 1. Introduction

The omics, such as genomics, transcriptomics, epigenomics, proteomics and metabolomics, have emerged, whereas the terms genome, transcriptome, epigenome, proteome and metabolome are used to address the objects of such studies, respectively [1,2,3,4,5]. The metabolome is a complete set of small-molecule types, such as endogenous intermediates, metabolism products or metabolites that are applied by metabolomics to study the response of biological systems, where metabolites are the final products of cellular regulatory processes [6].

Currently, the applications of metabolomics have increased to measure metabolic responses dynamically, identify biologically relevant candidate metabolic markers, reveal potential genetic architecture and understand the systematic networks underlying the economic traits for cattle [7,8,9,10,11,12,13]. For example, potential metabolic biomarkers, pathways or networks were identified for milk protein yield (MPY) and feed efficiency traits in dairy cattle using serum and plasma samples [8,9]. Hippuric acid, nicotinamide and pelargonic acid out of 36 significant metabolites were identified to play the key roles in MPY metabolism [8], whereas α-ketoglutarate and succinic acid were found in the network of feed efficiency [9]. Meanwhile, the metabolomic signatures associated with residual feed intake (RFI) trait in beef cattle were also found using plasma, rumen fluid, muscle, liver, etc., samples [7,10,12,13], where the retinol metabolism pathway is considered to be associated with feed efficiency [12]. Furthermore, significant metabolites in different tissues, such as in liver (citrate, isocitrate, glucose-6-phosphate, nicotinamide adenine dinucleotide + hydrogen and creatine phosphate) and in muscle (choline, glycine, glycerol, malonate, glucose-6-phosphate and 3-hydroxybutyrate), were studied to reveal useful metabolic signatures for Nellore cattle [13].

Given the previous findings in different traits (e.g., production, reproduction, nutrition, health, welfare), it is essential to summarize the major results of metabolomic analysis for further research and applications in cattle, as well further ingratiation analysis with other omics. Therefore, this review aims to investigate the existing metabolomic studies by highlighting the results from five aspects in cattle, integrated by other omics studies, e.g., genomics, transcriptomics, epigenomics, microbiomics, etc., to understand the metabolic mechanisms underlying the economic traits in cattle and to provide useful information for further cattle research and practical breeding programs.

## 2. Characterizations of Metabolomic Profiles in Cattle

The diversities of metabolome characterization occurring in cattle depend on the different breeds, traits, tissues, times, etc. To generate a better understanding of the underlying metabolic mechanisms in cattle, candidate metabolic biomarkers for various tissues and their enriched metabolic pathways are summarized in this review for important economic traits, such as feed efficiency and disease.

### 2.1. Candidate Metabolic Biomarkers for Various Tissues Associated with Production and Healthy Traits in Cattle Identified by Previous Studies

Based on previous studies, we found that researchers investigated the metabolomics of plasma, serum, milk, rumen fluid for feed efficiency, body performance, disease, etc., traits in cattle (Table 1). Feed efficiency is an important trait to produce more per feed that can be measured by gross feed efficiency (GFE), feed conversion ratio (FCR) or RFI methods [14,15]. Archer et al. (1999) [16] demonstrated that the inherent metabolic differences between animals can be reflected by the differences of RFI, while the RFI variation is underpinned by a combination of factors including metabolism [17]. Table 1 presents 24 metabolites that have been identified to be related to RFI, where citrate and succinic acid were repeatedly detected by several studies [7,9,10]. In addition, some studies found 1,3-dihydroxyacetone in association with fat, lactose and somatic cell score [18], but lysine and succinate in association with growth trait and feed efficiency traits [7,9,10] (Table 1).

Metabolic disease is another important trait that affects efficient cattle production, where metabolomic applications are helping to understand the mechanisms and define the predictive metabolic biomarkers for incident diseases [19]. Many metabolomic studies are revealing the associated metabolites with such diseases (Table 1); for instance, β-hydroxybutyrate is found to be mainly related to cattle disease traits that cause milk problems [20,21,22], because its concentration in blood is the main reason for hyperketonemia, which can be used as the disease diagnosis [23]. Benedet et al. (2019) [23] suggested that the thresholds of β-hydroxybutyrate concentration could be divided into three categories: ≤1.2 mmol/L as hyperketonemia indication; 1.2–2.9 mmol/L as subclinical ketosis; ≥3.0 mmol/L as clinical ketosis based on the other suggestions [24,25,26,27,28].

**Table 1:** Summary of candidate metabolic biomarkers associated with production and health traits in cattle identified by the previous studies.

Cattle	Trait	Sample Source	Metabolic Biomarker	Reference
Dairy cows (n = 1044)	Left displaced abomasum	Serum	β-hydroxybutyrate, Non-esterified fatty acids	LeBlanc et al. (2005) [20]
Holstein cows (n = 2356)	Early lactation milk loss	Serum	Non-esterified fatty acids, β-hydroxybutyrate	Chapinal et al. (2012) [22]
Holstein cows (n = 8)	Barley grain diet	Rumen fluid	Phenylalanine, Ornithine, Lysine, Leucine, Arginine, Valine, Phenylacetylglycine	Saleem et al. (2012) [29]
Danish Holstein and Jersey cows (n = 892)	Somatic cell count	Milk	β-hydroxybutyrate, Acetate, Butyrate, Fumarate, Hippurate, Isoleucine, Lactate	Sundekilde et al. (2012) [21]
Holstein cows (n = 1305)	Fat content	Milk	1,3-Dihydroxyaceton, Arabitol, Aspartic acid, Galactitol, Glucaric acid-1,4-lactone, Myo-Inositol-1-phosphate, Pyroglutamic acid	Melzer et al. (2013) [18]
Holstein cows (n = 1305)	pH value	Milk	β-Alanine, Glycerol-2-phosphate, Glycerol-3-phosphate, Glycine	Melzer et al. (2013) [18]
Holstein cows (n = 1305)	Protein content	Milk	Myo-Inositol-1-phosphate, Phosphoenolpyruvic acid, Pyroglutamic acid, Spermidine, 4-methyl-5-hydroxyethyl-Thiazole	Melzer et al. (2013) [18]
Holstein cows (n = 1305)	Lactose	Milk	1,3-Dihydroxyacetone, Glucaric acid-1,4-lactone, Leucine, Methionine, Phenylalanine, Tyrosine	Melzer et al. (2013) [18]
Holstein cows (n = 1305)	Milk quantity	Milk	Arabitol, 2-amino-Butanoic acid, 4-methylthio-2-oxo-Butanoic acid, 2-Piperidinecarboxylic acid	Melzer et al. (2013) [18]
Holstein cows (n = 1305)	Somatic cell score	Milk	1,3-Dihydroxyacetone, 2-hydroxy-Butanoic acid, Lactic acid, Leucine, Methionine, Phenylalanine, Tryptophan, Tyrosine, Uracil	Melzer et al. (2013) [18]
Holstein cows (n = 20)	Energy balance	Milk & Serum	Unsaturated fatty acids, Galactose-1-phosphate, Cholesterol, Stomatin	Lu et al. (2013) [30]
Holstein cows (n = 28)	Hepatic lipidosis	Serum	Glutamine, Glycine, Phosphatidyl-cholines, Sphingomyelins, Hydroxy-sphingomyelins	Imhasly et al. (2014) [31]
Crossbred beef cattle (Angus, Simmental, etc.) (n = 112)	Residual feed intake	Plasma	Acetate, Betaine, Carnitine, Citrate, Creatine, Formate, Glutamate, Glycine, Hippurate, Hydroxyisobutyrate, Lysine, Phenylalanine, Threonine, Tyrosine	Karisa et al. (2014) [7]
Crossbred beef cattle (Angus, Simmental, etc.) (n = 112)	Average daily gain	Plasma	Choline, Glutamate, Hippurate, Isoleucine	Karisa et al. (2014) [7]
Crossbred beef cattle (Angus, Simmental, etc.) (n = 112)	Average feed intake	Plasma	Acetate, Dimethyglycine, Glycerol, Glycol, Hippurate, Hydroxyisobutyrate, Lysine, Propylene, Succinate, Tyrosine	Karisa et al. (2014) [7]
Crossbred beef cattle (Angus, Simmental, etc.) (n = 112)	Average body weight	Plasma	Acetone, Formate, Glycerol, Hippurate, Hydroxyisobutyrate, Isopropanol, Lysine, Phenylalanine, Lysine	Karisa et al. (2014) [7]
Holstein calves (n = 12)	Systemic immune response	Plasma	Glycocholic acid, Glycine, Uric acid, Biliverdin, Taurodeoxycholic acid, Propionylcarnitine	Gray et al. (2015) [32]
German Holstein cows (n = 26)	Metabolic transition	Serum	Acylcarnitines, Glycerophospholipids, Sphingolipids	Kenéz et al. (2016) [11]
Simmental cows (n = 18)	Subacute rumen acidosis	Serum	Non-esterified fatty acids	Aditya et al. (2018) [33]
Holstein cows (n = 40)	Milk protein yield	Serum	Total cholesterol, Malonaldehyde	Wu et al. (2018) [8]
Danish Holstein and Jersey cows (n = 20)	Residual feed intake	Plasma	α-ketoglutarate, Succinic acid	Wang and Kadarmideen (2019) [9]
Beef steers (n = 29)	Residual feed intake	Rumen fluid	3,4-dihydroxyphenylacetate, 4-pyridoxate, Citraconate, Hypoxanthine, Succinate/Methylmalonate, Thymine, Xylose	Clemmons et al. (2020) [10]
Nellore and Angus beef cattle (n = 30)	Beef tenderness	Meat	Acetyl-carnitine, Adenine, Beta-alanine, Fumarate, Glutamine, Valine	Antonel et al. (2020) [34]

### 2.2. Revealed Metabolic Pathways in Cattle

**Figure 1:**
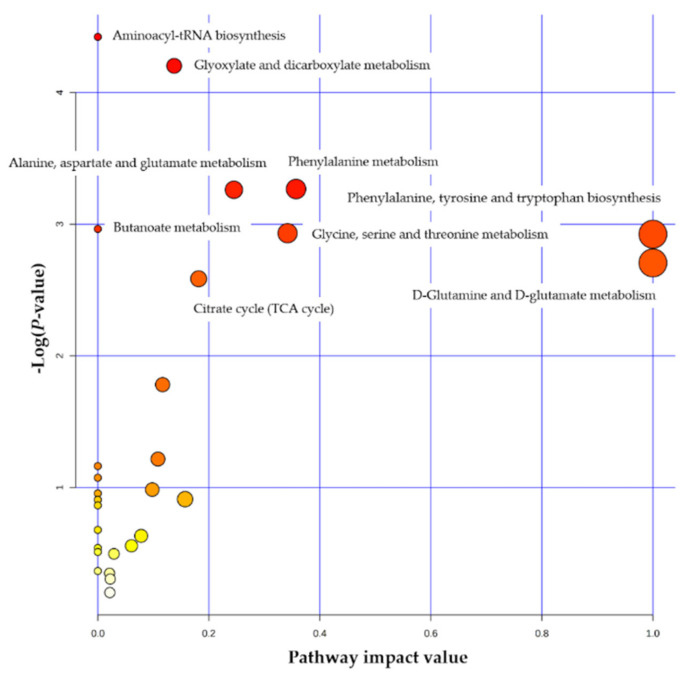
Pathway analysis for the metabolites associated with feed efficiency using *Bos taurus* as the library. Note: the pathway impact value is calculated using the sum of importance measures of the matched metabolites divided by the sum of the importance measures of all metabolites. The sizes and colors of the circles indicate the matched metabolite ratio and the log (*p*-value) of each pathway, respectively.

For the feed efficiency trait, the enrichment of the retinol metabolic pathway was revealed in beef cattle, where two metabolites in the pathway (a higher level of retinal and a lower level of retinoate) were found in the low feed efficient animals [12]. However, three important pathways that are the aminoacyl-tRNA biosynthesis, the alanine, aspartate, and glutamate metabolism, and the citrate cycle (TCA cycle) pathways were also associated with RFI in dairy cows using two types of pathway analysis [9]. In this review, we used the metabolites associated with RFI (n = 24, Table 1) to conduct the over-representation analysis (ORA) for metabolic pathway analysis. Fishers’ exact test for ORA was done by *MetaboAnalyst* software (version 5.0) [35], and metabolic pathways using the *Bos taurus* library were also realized to show the relative betweenness centrality against pathway impact value. The results showed that nine significantly metabolic pathways (FDR < 0.05) were revealed (Figure 1 and Supplementary Table S1), where the most significantly metabolic pathway was the aminoacyl-tRNA biosynthesis, followed by the glyoxylate and dicarboxylate metabolism and the phenylalanine metabolism (Figure 1). Six metabolites (glutamate, glycine, lysine, phenylalanine, threonine and tyrosine) were enriched in the aminoacyl-tRNA biosynthesis pathway (Supplementary Table S1) and the metabolite connections in the pathway were visualized in Supplementary Figure S1 using *MetaboAnalyst* software (version 5.0) [35].

**Figure 2:**
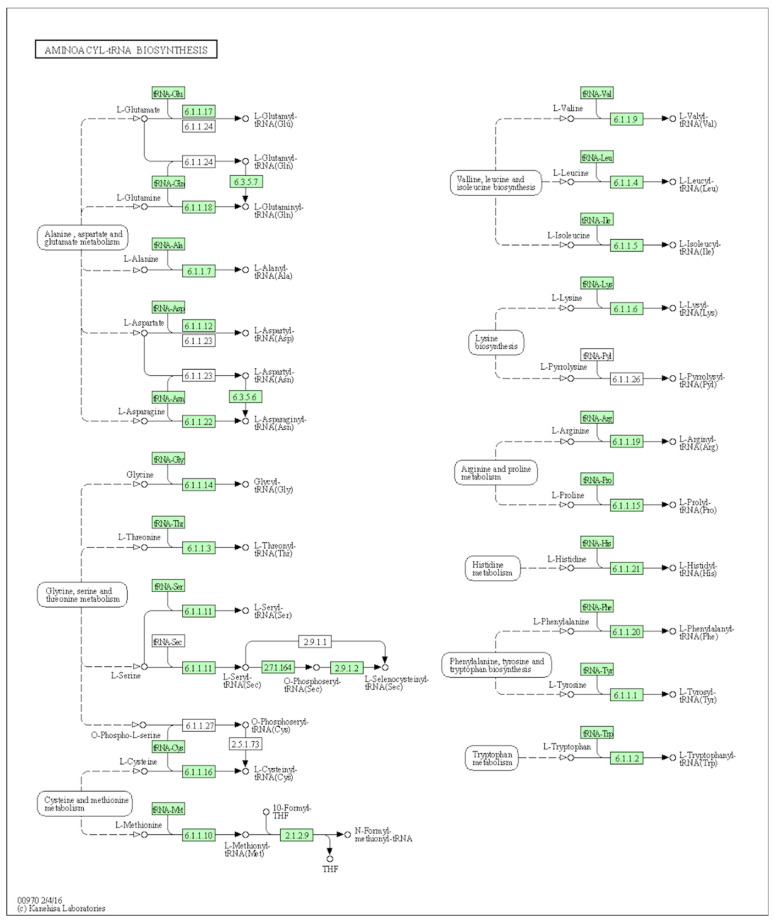
The mechanisms of aminoacyl-tRNA biosynthesis pathway (bta00970).

The aminoacyl-tRNA biosynthesis pathway is an amino acid metabolism and biosynthesis related pathway that has been identified as associated with RFI in dairy cows [9] and pigs [36]. This pathway is essential for normal growth and protein synthesis, and potentially influences cellular physiology and development [37,38]. Alanine, aspartate and glutamate metabolism, and the citrate cycle (TCA cycle)) pathways are also reported in relationship with feed efficiency traits [9], whereas the alanine, aspartate and glutamate metabolism is more sensitive to the diets and breed to affect the beef tenderness and meat sensory acceptability [34,39]. The mechanism illustration of the aminoacyl-tRNA biosynthesis pathway (bta00970) is shown in Figure 2, which is derived from the KEGG pathway database (https://www.genome.jp/kegg/, accessed on 20 September 2021) of *Bos taurus* species. It is suggested that the aminoacyl-tRNA biosynthesis pathway is mainly related to the other nine pathways. They are the alanine, aspartate and glutamate metabolism (bta00250), the glycine, serine and threonine metabolism (bta00260), the cysteine and methionine metabolism (bta00270), the valine, leucine and isoleucine biosynthesis (bta00290), the lysine biosynthesis (bta00300), the arginine and proline metabolism (bta00330), the histidine metabolism (bta00340), the phenylalanine, tyrosine and tryptophan biosynthesis (bta00400), and the tryptophan metabolism (bta00380) (Figure 2). It was found that the alanine, aspartate and glutamate metabolism was in a close relationship with the aminoacyl-tRNA biosynthesis pathway in terms of the mechanisms of feed efficiency regulation via the alanine, aspartate and glutamate metabolites [9].

## 3. Applications of Metabolomics in Cattle

Metabolomics has been applied in metabolic biomarker identification, genetic mechanism revelation, genomic prediction, understanding nutritional physiology, etc., for different economic traits of different species, which promotes the applications of metabolomics in cattle.

### 3.1. Revealed Biologically Genetic and Metabolic Related Mechanisms

The application of metabolomics and the other integrated omics data analysis lead to the clear cognition of the complex metabolic mechanisms [40]; for example, metabolome diversification occurs during different lactations [41,42]. Sun et al. (2017) [41] revealed five functionally enriched pathways (gluconeogenesis, pyruvate metabolism, TCA cycle, glycerolipid metabolism and aspartate metabolism) and suggested the TCA cycle, the glutamate metabolism, and the glycine biosynthesis and degradation pathways as the potential key metabolic mechanisms of lactation in the mammary gland. In fact, the aminoacyl-tRNA biosynthesis, the alanine, aspartate and glutamate metabolism, and the TCA cycle pathways also play key roles in the biochemical mechanisms in feed efficiency underlying metabolic biomarker variations (Table 1 and Figure 1). Most importantly, Wang and Kadarmideen [9] demonstrated one gene-metabolite network involved in the TCA cycle as the potential mechanism for RFI that modulates protein synthesis and regulates energy metabolism [43,44,45,46].

### 3.2. Improved Genomic Prediction for Complex Traits

Metabolomic-based genomic prediction has been conducted in plant species, such as wheat and barley, to display the potentiality of metabolite application as the predictor variables when no genotype is available [47,48,49,50]. Gemmer et al. (2020) [47] used the box-cox power method [51] to transform the metabolic data and then designed three prediction scenarios that are genomic prediction, metabolic prediction and the combined genomic-metabolic prediction. They found that both single-nucleotide polymorphisms (SNPs) and metabolites in the combined prediction scenario produced similar predictive abilities compared to the pure genomic prediction [47], which is consistent with other studies [48]. Nevertheless, Tong et al. (2020) [49] and Guo et al. (2016) [50] still found the integration of metabolites with genotypes significantly improved the prediction accuracies in maize and Arabidopsis, respectively; however, such predictive abilities were trait specific, so the metabolic information is suggested for use as predictors but to predict those traits directly related to metabolism. In animal breeding programs, useful metabolic information has also been suggested for incorporation into genomic prediction models or to be integrated with phenotypes or to be considered as the alternative phenotypes [52,53].

### 3.3. Understood Nutritional Biochemical Physiologies

Diet-based rumen metabolomic analysis can help reveal the nutritional biochemical physiology after feeding different diets [29,54]. For instance, when increasing proportions of barley grain diets were fed to dairy cows, metabolites (glucose, alanine, maltose, propionate, uracil, valerate, xanthine, ethanol, and phenylacetate) and methylamine concentrations in rumen increased as well, but the amount of 3-phenylpropionate decreased [54]. Similarly, Saleem et al. (2012) [29] explained more than 30% of grain diets influencing the health of dairy cattle because the rumen toxic or inflammatory fluid concentrations increased, such as putrescine, methylamines, ethanolamine and short-chain fatty acids. Different cattle feeding systems (e.g., only perennial ryegrass, total mixed ration and perennial ryegrass/white clover sward) could cause different metabolome profiles in milk and the subsequent products, such as amino acid composition in milk, and metabolome in skim milk and whey powders [55]. Sometimes, significant metabolome changes at different ages were found to indicate the identified metabolites as the potential biomarkers for early growing and fattening animals. Jeong et al. (2019) [56] revealed 19 metabolites and 3 metabolic pathways in beef cattle that assisted in a better understanding of cattle growth physiology for appropriate feeding strategies.

## 4. Integrated Metabolomic Analysis with Other Omics

Metabolomic analysis integrated with other omics data could contribute to the better understanding of the metabolomic complexity based on systems biology, but multiple layer integration would cause the challenge of statistics under the appropriate hypothesis [57,58]. The current integration analysis primarily focuses on two-layer interplays for the direct associations between two omics data that can be used to identify relevant candidate biomarkers, such as SNPs, genes, proteins, cytosine and guanine dinucleotides (CpGs), microbial communities, lipids, etc. It includes genomic−metabolomic analysis, transcriptomic/proteomic−metabolomic analysis, epigenomic−metabolomic analysis, microbiomic−metabolomic analysis and lipidomic−metabolomic analysis.

### 4.1. Genomics-Metabolomic Analysis

Metabolomics is the joint to connect genotypes with phenotypes [6], so their relationships are currently interpreted by the metabolome genome-wide association study (mGWAS) using metabolites as the metabolic phenotypes. The integrated genomic−metabolomic analysis is considered as a critical supplement to biology and physiology, as the metabolites provide the details of physiological state that can drive genetic variant-associated metabolites to display larger effect sizes, and then the quantitative trait loci (QTLs) affecting metabolite concentrations can be identified [53,59,60,61,62,63].

The genome-metabolite network has been constructed in bacterial species [59,60]; for example, large-scale metabolic models of *i*JL463 and *i*DZ470 were constructed for *Riemerella anatipestifer* wild type strain CH-1 (RA-CH-1, serotype 1) and *Riemerella anatipestifer* wild type strain CH-2 (RA-CH-2, serotype 2), respectively [59]. In beef cattle, Li et al. (2020) [61] detected three significant SNP associations (rs109862186, rs81117935 and rs42009425) for betaine, l-alanine and l-lactic acid, respectively; in addition, Wang and Kadarmideen (2020) found 152 genome-wide significant SNPs associated with 17 metabolites in pigs [53]. System biology analysis based on mGWAS can unravel significant SNPs related genes associated with metabolites and phenotypes. At the onset of puberty, Widmann et al. (2013) [62] found that Gonadotropin-releasing hormone (GnRH) signaling is associated with divergent growth in cattle.

The mGWAS is the direct association model between genomics and metabolomics to test the candidate SNPs or QTLs related to metabolites. It can be analyzed in the tools that are applied for GWAS, such as *EMMAX* (efficient mixed-model association eXpedited), *FaST-LMM* (factored spectrally transformed linear mixed models), *GCTA* (genome-wide complex trait analysis), *GEMMA* (genome-wide efficient mixed-model association) [64,65,66,67]. The mixed model is generally described as follows:

(1) y=Wa+Xb+Zg+e,

where y is the vector of phenotypes (e.g., metabolite values), W is the design matrix of covariates for fixed effects (e.g., breed, RFI, PCAs for genomic control [65,66,68,69]), a is the vector of fixed effects (i.e., corresponding coefficients) including the intercept, X is the marker covariates (i.e., SNP indicators 0, 1 or 2), b is the additive effect (fixed effect) of each marker to be tested, Z is the design matrix for g, g is the vector of polygenic effects as random effects that are the accumulated effects of all markers (i.e., captured by genetic relationship matrix (GRM) calculated using all SNPs) and e is the vector of residual effects. The polygenic and residual variances are $\mathrm{Var}[g]={\mathrm{Go}}_{g}^{2}$ and $\mathrm{Var}[e]={I\sigma }_{e}^{2}$, where G and I are the GRM and identity matrix, respectively.

### 4.2. Transcriptomic-Metabolomic Analysis

The gene-metabolite interplay network can be constructed for transcriptomic-metabolomic analysis based on the Kyoto Encyclopedia of Genes and Genomes (KEGG) pathways using *MetaboAnalyst* [70,71]; for example, one gene (2-hydroxyacyl-CoA lyase 1 (*HACL1*)) associated with two metabolites (α-ketoglutarate and succinic acid) was identified in high-low feed efficient dairy cattle [9]. Likewise, web tools *IMPaLA*, *Metabox* (R based), *XCMS*, etc., [72,73,74,75] also integrate metabolomic data with transcriptomics on the pathway level. Interactions between genes and metabolites in different combinations of biological networks can enhance our knowledge of underlying biological mechanisms by reflecting the cellular regulations in different layers [72,73]. Based on the BioCyc [76], KEGG [77] and Uniprot [78] databases, the genes and proteins can be mapped on the predicted metabolic pathways [73].

R package *IntLIM* [79] was used to integrate metabolomics and gene expression data for feed efficiency traits in pigs [80], where the interactions of phenotypes and gene expressions are fitted in the model [79]. The linear model that *IntLIM* [79] used is as follows:

(2) $$m=\beta_{1}+\beta_{2}g+\beta_{3}p+\beta_{4}\left(g:p\right)+\varepsilon ,$$

where m, g and p are metabolite values (normalized), gene expression levels (log2-transformed) and phenotypes (case-control designed), respectively. Here, g:p represents the statistical interaction between gene expressions and experimental designed phenotypes, where a significant two-tailed *p*-value indicates the gene-metabolite association is different from the cases to the controls [79,81].

### 4.3. Other Two-Layer Omics−Metabolomic Analysis

For the two-layer omics data integration, epigenomic−metabolomic interactions could discover novel molecular targets via epigenetic mechanisms regulating the expression levels of metabolic genes and thereby altering the metabolome [82,83]. Wong et al. (2017) [82] suggested that epigenetic drugs (e.g., DNMT and HDAC inhibitors) could be used to target metabolic reprogramming in cancer cells. In their review, they also considered the combination of metabolism inhibitors and epigenetic modulators to achieve synergistic tumor inhibition as the developmental approach [82]. On the other hand, Petersen et al. (2014) [83] conducted an epigenome-wide association study for blood serum metabolites to investigate the relationship between DNA methylation and metabolic traits. They found that the underlying genetic effects or environmental effects mainly drove the methylome–metabotype associations, and identified several CpG site-specific associations with metabolites; therefore, DNA methylation has an important role in regulating the metabolism [83].

So far, the analysis between microbiome and metabolome could predict which compounds have been produced by a community of bacteria or the host in an R package *AMON* [84]. However, another similar web tool *MIMOSA* [85] is a relatively quantitative tool that determines the quantitative relationships between the relative abundance of genes in a metagenome and the abundance of the particular compounds in a metabolome. Moreover, Mallick et al. (2019) [86] developed the MelonnPan algorithm to predict the unobserved metabolite features in the new microbial communities by incorporating biological knowledge.

Lipidome is one subset of the metabolome as same to amino acids, sugars and nucleic acids, but lipidomics has emerged as an independent field due to the functionally structural diversity and high endogenous abundance of lipids resulting in the complexities of the organismal lipidomes [87,88]. The integration analysis between metabolomics and lipidomics are normally applied to understand the cellular mechanism and to reveal signatures for human diseases [88,89]. Wang et al. (2019) [88] summarized the previous studies on the roles of lipids and metabolites for diseases, and found that the integrated analysis of metabolomics and lipidomics was critical for the revelation of cellular biology and disease pathology. Acharjee et al. (2016) [89] used a machine learning approach to integrate the metabolomics, lipidomics and clinical data. They pinpointed that lipidomics was the most predictive data responding to different doses and then established the relationships of the metabolic and lipidomic data with aspartate amino transaminase [89].

### 4.4. Multiple Integrated Omics−Metabolomic Analysis

In the previous study, transcriptomic, proteomic and metabolomic integrated analysis was used to investigate the overexpression and inhibition of miR-223 affecting gene regulation in the cytoplasm of the monocyte−macrophage cell line [90]. They characterized the three-layer integrated metabolomic analysis with other omics responses to miR-223 modulation, and found that the miR-223 alteration changed the gene expressions (*CARM-1*, *Ube2g2*, *Cactin* and *Ndufaf6*) during macrophage differentiation and osteoclastogenesis and the metabolic profile of cells to potentially influence the apoptotic and proliferative states [90]. Jamil et al. (2020) [91] also proposed three levels of integration analysis for transcriptomic−proteomic−metabolomic data that are element-based (e.g., correlation and clustering), pathway-based (e.g., pathway and co-expression) and more complex mathematical-based levels. Frau et al. (2019) [92] firstly integrated metabolome, microbiome and mycobiome data in Crohn’s disease (CD) with the aim of investigating the correlation of fungi metabolites with fungal species in CD patients; finally, they understood which microorganisms were likely active in CD and which microorganisms produced the metabolites of interest.

## 5. Methods and Tools Applied in Metabolomics Analysis

The current metabolomics analysis methods and tools are widely applied for metabolic biomarker detection, cluster classification, pathway and network identification, two-layer data integration, etc., which is usually limited in the metabolomics category (Table 2). Statistical methods and analyzing tools for multiple-layer integrations are still necessary for the further integrated metabolomics analysis with other omics. The important features and used environments of the current tools are listed in Table 2.

Generally, a linear regression model is considered to analyze metabolomics data for significant metabolite identification by fitting phenotypes (e.g., RFI) as covariates [9,80]. Sometimes, the elastic net regularization model is also applied to fit microbial communities [86]. The analysis tools can be in a web environment or be directly used by the related R packages, such as *MetabR*, *glmnet*, *IntLIM*, etc., [79,93,94].

In order to cluster the metabolites, principal component analysis (PCA), linear discriminant analysis (LDA) and partial least squares discriminant analysis (PLS-DA) are normally used in the R packages or other tools, such as *MetaboAnalyst*, *VOCCluster* [93,95]. Tools with new features, such as interactive time-series cluster analysis (R package *MetaboClust*) [96], automated hierarchical cluster (R package *hcapca*) [97] are also developed for clustering analysis. Bayesian network method (BNM) can model the interactions of the metabolites to identify important metabolites in the optimal network, which has been demonstrated in the study of Rogers et al. (2014) [98]. The predictive accuracy of BNM with an area under the curve convex hull (AUCCH) was higher than PLS-DA, as PLS-DA probably led to overfitting that was indicated by the permutation test [99].

Notably, metabolomics data is complex and nonlinear, so machine-learning methods are applied for the nonlinear data interpretation based on random forest, support vector machine, artificial neural network algorithms, etc., [100,101,102,103,104]. For example, Ghaffari et al. (2019) [105] employed the machine-learning methods to reveal 12 significant metabolites and 2 meaningful pathways in normal versus over-conditioned cows. Such methods with the developed tools can also be used for biomarker detection, classification, biochemical pathway identification and multi-omics integration [103].

Some transcriptomic expression and co-expression analysis approaches are also available for metabolic data by considering the metabolite values as the expression levels to perform the metabolite analysis and the interacted networks, such as using R package *limma* and *WGCNA* [106,107,108,109,110]. *WGCNA* (weighted correlation network analysis) can construct a similarity matrix by Pearson correlation coefficients to measure the profiles’ similarity for the further network construction, and then identify the metabolically relevant key modules [107].

**Table 2:** Metabolomics analysis tools and their features.

Analysis Tool	Environment	Feature	Reference
WGCNA	R	Weighted correlation network analysis	Langfelder et al. (2008) [107]
MetaboAnalyst	Web/R	Statistical, biomarker, pathway, joint-pathway, network, meta-analysis, etc.	Xia et al. (2009) [93]/Xia et al. (2012) [111]/Xia and Wishart (2016) [112]/Chong and Xia (2018) [113]/Chong et al. (2019) [71]
glmnet	R	Statistical analysis in lasso or elastic net model	Friedman et al. (2010) [114]
MetabR	R	Statistical analysis in linear model	Ernest et al. (2012) [94]
muma	R	Step-wise pipeline for metabolomics univariate and multivariate statistical analyses	Gaude et al. (2013) [115]
limma	R	Statistical analysis in linear model by considering the metabolite values as the expression data	Ritchie et al. (2015) [110]
MetabNet	R	Targeted metabolome-wide association study for pathway and network mapping	Uppal et al. (2015) [116]
MIMOSA	Web	Quantitative relationships between the relative abundance of genes in a metagenome and the abundance of the particular compounds in a metabolome	Noecker et al. (2016) [85]
IntLIM	R	Integration analysis of transcriptomic and metabolomic data	Siddiqui et al. (2018) [79]
MetaboClust	R	Interactive time-series cluster	Rusilowicz et al. (2018) [96]
MetaboDiff	R	Exploration of sample traits in a data-derived metabolic correlation network	Mock et al. (2018) [117]
NormalizeMets	R	Visualisation of metabolomics data using interactive graphical displays and to obtain end statistical results for clustering, classification, biomarker identification adjusting for confounding variables, and correlation analysis	de Livera et al. (2018) [118]
AMON	R	Prediction of compounds that could have been produced by community of bacteria or the host	Shaffer et al. (2019) [84]
MelonnPan	R	Unobserved metabolite feature prediction in new microbial communities by incorporating biological knowledge	Mallick et al. (2019) [86]
hcapca	R	Automated hierarchical cluster	Chanana et al. (2020) [97]
MetaboShiny	R	Database- and formula-prediction-based annotation and visualization for mass spectrometry data	Wolthuis et al. (2020) [119]
MetENP	Web/R	Species-specific pathway analysis, pathway enrichment scores, gene-enzyme information, and enzymatic activities of the significantly altered metabolites	Choudhary et al. (2020) [120]
VOCCluster	R	Untargeted feature cluster using gas chromatography/mass spectrometry (GC/MS) data	Alkhalifah et al. (2020) [95]

## 6. Implications and Further Potentialities

**Figure 3:**
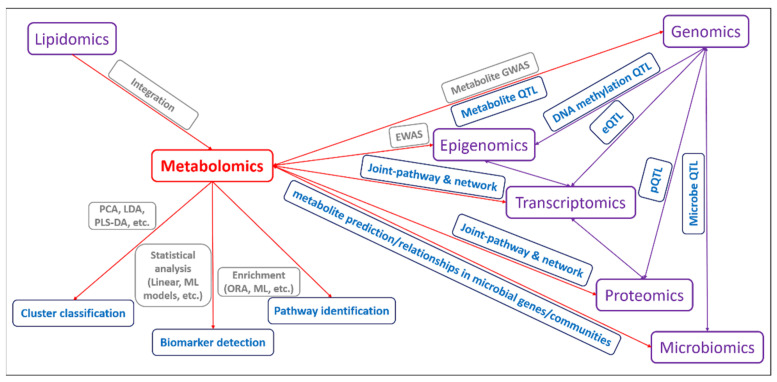
Metabolomics analysis workflow and the integrated analysis with other omics data in cattle.

Metabolomic and integrated other omics data analysis are available for the measurement of dynamically metabolic responses, identification of biologically metabolic markers, revelation of potentially genetic architecture, understanding of systematic networks. In cattle, the classified metabolome clusters, detected relevant candidate metabolites, identified metabolic pathways, potential systematic networks have been studied to achieve promising and meaningful results. We summarized the previous results of one-layer metabolomic analysis and potential two-layer integration analysis that can be presented in Figure 3, as discussed above. We fully believe that the summarized results are useful for metabolomic application in cattle farms.

Multiple integrated omics analysis would become critical and favorable in a further study, especially for the integration of genomics, epigenomics, transcriptomics, proteomics, microbiomics and lipidomics to reveal the metabolic-related mechanisms, identify multiple-layer biomarkers and improve genomic predictions, etc. Theoretically, metabolite QTLs, DNA methylation QTLs, expression QTLs (eQTLs), protein QTLs (pQTLs), microbe QTLs could be integrated on the genome level by constructing a multiple-omics network using those QTLs as the joint. In the meantime, the annotated genes/proteins to QTLs affecting metabolite concentrations that are regulated by the genetic/epigenetic variants could be connected together based on the biological pathways (Figure 3). Thus, the network construction of genomics, epigenomics, transcriptomics, proteomics, metabolomics with the joints from the genomic level to the pathway level is derived, but possibly four-layer integrations (QTLs−genes/proteins (CpG regulated) −pathways−metabolites) are difficult and challenging to find out or to identify by the current relatively smaller sample sizes, so larger populations are still necessary for further validation in cattle using one promising economic trait.

## 7. Conclusions

In summary, this review concludes the useful metabolic information from the previous research results including the characterizations of metabolomics profiles, metabolomics applications, integrated metabolomics analysis with other omics, methods and tools in metabolomics analysis, and the further potentialities and implications in cattle, which may contribute to the production improvement, disease reduction, efficient farming for the cattle economically important traits.

## Supplementary Materials

The following are available online at www.mdpi.com/article/10.3390/metabo11110753/s1, Supplementary Table S1: Significantly metabolic pathways (FDR < 0.05) for the metabolites associated with feed efficiency using *Bos taurus* as the library. Supplementary Figure S1: The connections of the metabolites including glutamate, glycine, lysine, phenylalanine, threonine and tyrosine (red color) enriched in the aminoacyl-tRNA biosynthesis pathway.

## References

[1] Hasin Y., Seldin M., Lusis A. (2017). Multi-omics approaches to disease. Genome Biol..

[2] Wasinger V., Cordwell S., Poljak A., Yan J.X., Gooley A.A., Wilkins M.R., Duncan M., Harris R., Williams K.L., Humphery-Smith I. (1995). Progress with gene-product mapping of the mollicutes: *Mycoplasma genitalium*. Electrophoresis.

[3] Vailati-Riboni M., Palombo V., Loor J.J. (2017). What are omics sciences?. Periparturient Diseases of Dairy Cows: A Systems Biology Approach.

[4] Oliver S.G. (1998). Systematic functional analysis of the yeast genome. Trends Biotechnol..

[5] Schena M., Shalon D., Davis R.W., Brown P.O. (1995). Quantitative monitoring of gene expression patterns with a complementary DNA microarray. Science.

[6] Fiehn O. (2002). Metabolomics—The link between genotypes and phenotypes. Plant Mol. Biol..

[7] Karisa B., Thomson J., Wang Z., Li C., Montanholi Y., Miller S., Moore S., Plastow G. (2014). Plasma metabolites associated with residual feed intake and other productivity performance traits in beef cattle. Livest. Sci..

[8] Wu X., Sun H., Xue M., Wang D., Guan L.L., Liu J. (2018). Serum metabolome profiling revealed potential biomarkers for milk protein yield in dairy cows. J. Proteom..

[9] Wang X., Kadarmideen H.N. (2019). Metabolomics analyses in high-low feed efficient dairy cows reveal novel biochemical mechanisms and predictive biomarkers. Metabolites.

[10] Clemmons B.A., Powers J.B., Campagna S.R., Seay T.B., Embree M.M., Myer P.R. (2020). Rumen fluid metabolomics of beef steers differing in feed efficiency. Metabolomics.

[11] Kenéz Á., Dänicke S., Rolle-Kampczyk U., von Bergen M., Huber K. (2016). A metabolomics approach to characterize phenotypes of metabolic transition from late pregnancy to early lactation in dairy cows. Metabolomics.

[12] Novais F.J., Pires P.R.L., Alexandre P.A., Dromms R.A., Iglesias A.H., Ferraz J.B.S., Styczynski M.P.-W., Fukumasu H. (2019). Identification of a metabolomic signature associated with feed efficiency in beef cattle. BMC Genom..

[13] Cônsolo N., Buarque V., Silva J., Poleti M., Barbosa L., Higuera-Padilla A., Gómez J., Colnago L., Gerrard D., Netto A.S. (2021). Muscle and liver metabolomic signatures associated with residual feed intake in Nellore cattle. Anim. Feed. Sci. Technol..

[14] Connor E. (2015). Invited review: Improving feed efficiency in dairy production: Challenges and possibilities. Animal.

[15] Koch R.M., Swiger L.A., Chambers D., Gregory K.E. (1963). Efficiency of feed use in beef cattle. J. Anim. Sci..

[16] Archer J.A., Richardson E.C., Herd R.M., Arthur P.F. (1999). Potential for selection to improve efficiency of feed use in beef cattle: A review. Aust. J. Agric. Res..

[17] Digiacomo K., Norris E., Dunshea F., Hayes B., Marett L., Wales W., Leury B. (2018). Responses of dairy cows with divergent residual feed intake as calves to metabolic challenges during midlactation and the nonlactating period. J. Dairy Sci..

[18] Melzer N., Wittenburg D., Hartwig S., Jakubowski S., Kesting U., Willmitzer L., Lisec J., Reinsch N., Repsilber D. (2013). Investigating associations between milk metabolite profiles and milk traits of Holstein cows. J. Dairy Sci..

[19] Newgard C.B. (2017). Metabolomics and metabolic diseases: Where do we stand?. Cell Metab..

[20] Leblanc S.J., Leslie K.E., Duffield T.F. (2005). Metabolic predictors of displaced abomasum in dairy cattle. J. Dairy Sci..

[21] Sundekilde U., Poulsen N., Larsen L.B., Bertram H. (2013). Nuclear magnetic resonance metabonomics reveals strong association between milk metabolites and somatic cell count in bovine milk. J. Dairy Sci..

[22] Chapinal N., Carson M., LeBlanc S., Leslie K., Godden S., Capel M., Santos J., Overton M., Duffield T. (2012). The association of serum metabolites in the transition period with milk production and early-lactation reproductive performance. J. Dairy Sci..

[23] Benedet A., Manuelian C.L., Zidi A., Penasa M., de Marchi M. (2019). Invited review: β-hydroxybutyrate concentration in blood and milk and its associations with cow performance. Animal.

[24] McArt J., Nydam D., Ospina P., Oetzel G. (2011). A field trial on the effect of propylene glycol on milk yield and resolution of ketosis in fresh cows diagnosed with subclinical ketosis. J. Dairy Sci..

[25] McArt J., Nydam D., Oetzel G. (2012). Epidemiology of subclinical ketosis in early lactation dairy cattle. J. Dairy Sci..

[26] Weigel K., Pralle R.S., Adams H., Cho K., Do C., White H. (2017). Prediction of whole--genome risk for selection and management of hyperketonemia in Holstein dairy cattle. J. Anim. Breed. Genet..

[27] Vanholder T., Papen J., Bemers R., Vertenten G., Berge A.C. (2015). Risk factors for subclinical and clinical ketosis and association with production parameters in dairy cows in the Netherlands. J. Dairy Sci..

[28] Rutherford A.J., Oikonomou G., Smith R.F. (2016). The effect of subclinical ketosis on activity at estrus and reproductive performance in dairy cattle. J. Dairy Sci..

[29] Saleem F., Ametaj B., Bouatra S., Mandal R., Zebeli Q., Dunn S., Wishart D.S. (2012). A metabolomics approach to uncover the effects of grain diets on rumen health in dairy cows. J. Dairy Sci..

[30] Lu J., Fernandes E.A., Cano A.E.P., Vinitwatanakhun J., Boeren S., van Hooijdonk T., van Knegsel A., Vervoort J., Hettinga K.A. (2013). Changes in milk proteome and metabolome associated with dry period length, energy balance, and lactation stage in postparturient dairy cows. J. Proteome Res..

[31] Imhasly S., Naegeli H., Baumann S., von Bergen M., Luch A., Jungnickel H., Potratz S., Gerspach C. (2014). Metabolomic biomarkers correlating with hepatic lipidosis in dairy cows. BMC Vet.-Res..

[32] Gray D.W., Welsh M.D., Doherty S., Mansoor F., Chevallier O.P., Elliott C.T., Mooney M.H. (2015). Identification of systemic immune response markers through metabolomic profiling of plasma from calves given an intra-nasally delivered respiratory vaccine. Vet. Res..

[33] Aditya S., Humer E., Pourazad P., Khiaosa-Ard R., Zebeli Q. (2018). Metabolic and stress responses in dairy cows fed a concentrate-rich diet and submitted to intramammary lipopolysaccharide challenge. Animal.

[34] Antonelo D., Gerrard D.E., Gómez J.F.M., Balieiro J.C., Colnago L.A., Beline M., Cônsolo N., Silva S.L., Suman S.P., Schilling W. (2020). Metabolites and metabolic pathways correlated with beef tenderness. Meat Muscle Biol..

[35] Xia J., Wishart D.S. (2010). MetPA: A web-based metabolomics tool for pathway analysis and visualization. Bioinformatics.

[36] Jiang H., Fang S., Yang H., Chen C. (2021). Identification of the relationship between the gut microbiome and feed efficiency in a commercial pig cohort. J. Anim. Sci..

[37] Kyriacou S.V., Deutscher M.P. (2008). An important role for the multienzyme aminoacyl-tRNA synthetase complex in mammalian translation and cell growth. Mol. Cell.

[38] Lu J., Bergert M., Walther A., Suter B. (2014). Double-sieving-defective aminoacyl-tRNA synthetase causes protein mistranslation and affects cellular physiology and development. Nat. Commun..

[39] Graham S.F., Kennedy T., Chevallier O., Gordon A., Farmer L., Elliott C., Moss B. (2010). The application of NMR to study changes in polar metabolite concentrations in beef longissimus dorsi stored for different periods post mortem. Metabolomics.

[40] Loor J.J., Bionaz M., Invernizzi G. (2011). Systems biology and animal nutrition: Insights from the dairy cow during growth and the lactation cycle. Systems Biology and Livestock Science.

[41] Sun H.-Z., Shi K., Wu X.-H., Xue M.-Y., Wei Z.-H., Liu J.-X., Liu H.-Y. (2017). Lactation-related metabolic mechanism investigated based on mammary gland metabolomics and 4 biofluids’ metabolomics relationships in dairy cows. BMC Genom..

[42] Garnsworthy P.C. (1988). Nutrition and Lactation in the Dairy Cow.

[43] Wu N., Yang M., Gaur U., Xu H., Yao Y., Li D. (2016). Alpha-ketoglutarate: Physiological functions and applications. Biomol. Ther..

[44] Tretter L., Patocs A., Chinopoulos C. (2016). Succinate, an intermediate in metabolism, signal transduction, ROS, hypoxia, and tumorigenesis. Biochim. Biophys. Acta BBA Bioenergy.

[45] Soghomonian J.-J., Martin D.L. (1998). Two isoforms of glutamate decarboxylase: Why?. Trends Pharmacol. Sci..

[46] Do D.N., Ostersen T., Strathe A.B., Mark T., Jensen J., Kadarmideen H.N. (2014). Genome-wide association and systems genetic analyses of residual feed intake, daily feed consumption, backfat and weight gain in pigs. BMC Genet..

[47] Gemmer M.R., Richter C., Jiang Y., Schmutzer T., Raorane M.L., Junker B., Pillen K., Maurer A. (2020). Can metabolic prediction be an alternative to genomic prediction in barley?. PLoS ONE.

[48] Zhao Y., Li Z., Liu G., Jiang Y., Maurer H.P., Würschum T., Mock H.-P., Matros A., Ebmeyer E., Schachschneider R. (2015). Genome-based establishment of a high-yielding heterotic pattern for hybrid wheat breeding. Proc. Natl. Acad. Sci. USA.

[49] Tong H., Küken A., Nikoloski Z. (2020). Integrating molecular markers into metabolic models improves genomic selection for Arabidopsis growth. Nat. Commun..

[50] Guo Z., Magwire M.M., Basten C.J., Xu Z., Wang D. (2016). Evaluation of the utility of gene expression and metabolic information for genomic prediction in maize. Theor. Appl. Genet..

[51] Box G.E.P., Cox D.R. (1964). An analysis of transformations. J. R. Stat. Soc. Ser. B Stat. Methodol..

[52] Fontanesi L. (2016). Metabolomics and livestock genomics: Insights into a phenotyping frontier and its applications in animal breeding. Anim. Front..

[53] Wang X., Kadarmideen H.N. (2020). Metabolite genome-wide association study (mGWAS) and gene-metabolite interaction network analysis reveal potential biomarkers for feed efficiency in pigs. Metabolites.

[54] Ametaj B.N., Zebeli Q., Saleem F., Psychogios N., Lewis M.J., Dunn S.M., Xia J., Wishart D.S. (2010). Metabolomics reveals unhealthy alterations in rumen metabolism with increased proportion of cereal grain in the diet of dairy cows. Metabolomics.

[55] Magan J.B., O’Callaghan T.F., Zheng J., Zhang L., Mandal R., Hennessy D., Fenelon M.A., Wishart D.S., Kelly A.L., McCarthy N.A. (2019). Impact of bovine diet on metabolomic profile of skim milk and whey protein ingredients. Metabolites.

[56] Jeong J.Y., Kim M., Reddy K.E., Lee S., Cho S., Lee H.-J. (2019). PSXI-12 comparative metabolomics of blood plasma from Hanwoo beef cattle at different ages and fed diets with different nutritional levels, by using liquid chromatography-mass spectrometry. J. Anim. Sci..

[57] Kadarmideen H. (2014). Genomics to systems biology in animal and veterinary sciences: Progress, lessons and opportunities. Livest. Sci..

[58] Suravajhala P., Kogelman L.J.A., Kadarmideen H.N. (2016). Multi-omic data integration and analysis using systems genomics approaches: Methods and applications in animal production, health and welfare. Genet. Sel. Evol..

[59] Liu J., Cheng A., Wang M., Liu M., Zhu D., Yang Q., Wu Y., Jia R., Chen S., Zhao X. (2021). Comparative genomics and metabolomics analysis of *Riemerella anatipestifer* strain CH-1 and CH-2. Sci. Rep..

[60] Planchon M., Léger T., Spalla O., Huber G., Ferrari R. (2017). Metabolomic and proteomic investigations of impacts of titanium dioxide nanoparticles on Escherichia coli. PLoS ONE.

[61] Li J., Akanno E.C., Valente T.S., Abo-Ismail M., Karisa B.K., Wang Z., Plastow G.S. (2020). Genomic heritability and genome-wide association studies of plasma metabolites in crossbred beef cattle. Front. Genet..

[62] Widmann P., Reverter A., Fortes M.R.S., Weikard R., Suhre K., Hammon H., Albrecht E., Kuehn C. (2013). A systems biology approach using metabolomic data reveals genes and pathways interacting to modulate divergent growth in cattle. BMC Genom..

[63] Gieger C., Geistlinger L., Altmaier E., de Angelis M.H., Kronenberg F., Meitinger T., Mewes H.-W., Wichmann H.-E., Weinberger K., Adamski J. (2008). Genetics meets metabolomics: A genome-wide association study of metabolite profiles in human serum. PLoS Genet..

[64] Zhou X., Stephens M. (2012). Genome-wide efficient mixed-model analysis for association studies. Nat. Genet..

[65] Kang H.M., Sul J.H., Service S.K., Zaitlen N.A., Kong S.-Y., Freimer N.B., Sabatti C., Eskin E. (2010). Variance component model to account for sample structure in genome-wide association studies. Nat. Genet..

[66] Yang J., Lee S.H., Goddard M.E., Visscher P.M. (2011). GCTA: A tool for genome-wide complex trait analysis. Am. J. Hum. Genet..

[67] Lippert C., Listgarten J., Liu Y., Kadie C.M., Davidson R.I., Heckerman D. (2011). FaST linear mixed models for genome-wide association studies. Nat. Methods.

[68] Bacanu S.-A., Devlin B., Roeder K. (2002). Association studies for quantitative traits in structured populations. Genet. Epidemiol..

[69] Devlin B., Roeder K. (1999). Genomic control for association studies. Biometrics.

[70] Chong J., Soufan O., Li C., Caraus I., Li S., Bourque G., Wishart D.S., Xia J. (2018). MetaboAnalyst 4.0: Towards more transparent and integrative metabolomics analysis. Nucleic Acids Res..

[71] Chong J., Wishart D.S., Xia J. (2019). Using MetaboAnalyst 4.0 for comprehensive and integrative metabolomics data analysis. Curr. Protoc. Bioinform..

[72] Wanichthanarak K., Fan S., Grapov D., Barupal D.K., Fiehn O. (2017). Metabox: A toolbox for Metabolomic data analysis, interpretation and integrative exploration. PLoS ONE.

[73] Huan T., Forsberg E.M., Rinehart D., Johnson C., Ivanisevic J., Benton H.P., Fang M., Aisporna A., Hilmers B., Poole F.L. (2017). Systems biology guided by XCMS online metabolomics. Nat. Methods.

[74] Kamburov A., Cavill R., Ebbels T., Herwig R., Keun H.C. (2011). Integrated pathway-level analysis of transcriptomics and metabolomics data with IMPaLA. Bioinformatics.

[75] Xia J., Fjell C., Mayer M.L., Pena O.M., Wishart D.S., Hancock R. (2013). INMEX—A web-based tool for integrative meta-analysis of expression data. Nucleic Acids Res..

[76] Caspi R., Billington R., Ferrer L., Foerster H., Fulcher C.A., Keseler I.M., Kothari A., Krummenacker M., Latendresse M., Mueller L.A. (2016). The MetaCyc database of metabolic pathways and enzymes and the BioCyc collection of pathway/genome databases. Nucleic Acids Res..

[77] Ogata H., Goto S., Sato K., Fujibuchi W., Bono H., Kanehisa M. (1999). KEGG: Kyoto encyclopedia of genes and genomes. Nucleic Acids Res..

[78] The UniProt Consortium (2019). UniProt: A worldwide hub of protein knowledge. Nucleic Acids Res..

[79] Siddiqui J.K., Baskin E., Liu M., Cantemir-Stone C.Z., Zhang B., Bonneville R., McElroy J.P., Coombes K.R., Mathé E.A. (2018). IntLIM: Integration using linear models of metabolomics and gene expression data. BMC Bioinform..

[80] Banerjee P., Carmelo V.A.O., Kadarmideen H.N. (2020). Integrative analysis of metabolomic and transcriptomic profiles uncovers biological pathways of feed efficiency in pigs. Metabolites.

[81] Boulesteix A.-L., Janitza S., Hapfelmeier A., van Steen K., Strobl C. (2015). Letter to the editor: On the term ’interaction’ and related phrases in the literature on random forests. Brief. Bioinform..

[82] Wong C.C., Qian Y., Yu J. (2017). Interplay between epigenetics and metabolism in oncogenesis: Mechanisms and therapeutic approaches. Oncogene.

[83] Petersen A.-K., Zeilinger S., Kastenmüller G., Römisch-Margl W., Brugger M., Peters A., Meisinger C., Strauch K., Hengstenberg C., Pagel P. (2014). Epigenetics meets metabolomics: An epigenome-wide association study with blood serum metabolic traits. Hum. Mol. Genet..

[84] Shaffer M., Thurimella K., Quinn K., Doenges K., Zhang X., Bokatzian S., Reisdorph N., Lozupone C.A. (2019). AMON: Annotation of metabolite origins via networks to integrate microbiome and metabolome data. BMC Bioinform..

[85] Noecker C., Eng A., Srinivasan S., Theriot C.M., Young V.B., Jansson J.K., Fredricks D., Borenstein E. (2016). Metabolic model-based integration of microbiome taxonomic and metabolomic profiles elucidates mechanistic links between ecological and metabolic variation. MSystems.

[86] Mallick H., Franzosa E.A., Mclver L.J., Banerjee S., Sirota-Madi A., Kostic A.D., Clish C.B., Vlamakis H., Xavier R.J., Huttenhower C. (2019). Predictive metabolomic profiling of microbial communities using amplicon or metagenomic sequences. Nat. Commun..

[87] Lam S.M., Tian H., Shui G. (2017). Lipidomics, en route to accurate quantitation. Biochim. Biophys. Acta BBA—Mol. Cell Biol. Lipids.

[88] Wang R., Li B., Lam S.M., Shui G. (2020). Integration of lipidomics and metabolomics for in-depth understanding of cellular mechanism and disease progression. J. Genet. Genom..

[89] Acharjee A., Ament Z., West J.A., Stanley E., Griffin J.L. (2016). Integration of metabolomics, lipidomics and clinical data using a machine learning method. BMC Bioinform..

[90] M’Baya-Moutoula E., Louvet L., Molinié R., Guerrera I.C., Cerutti C., Fourdinier O., Nourry V., Gutierrez L., Morlière P., Mesnard F. (2018). A multi-omics analysis of the regulatory changes induced by miR-223 in a monocyte/macrophage cell line. Biochim. Biophys. Acta BBA—Mol. Basis Dis..

[91] Jamil I.N., Remali J., Azizan K.A., Nor Muhammad N.A., Arita M., Goh H.H., Aizat W.M. (2020). Systematic multi-omics integration (MOI) approach in plant systems biology. Front. Plant Sci..

[92] Frau A., Hough R., Ijaz U., Campbell B., Kenny J., Hall N., Anson J., Darby A., Probert C. (2019). Metabolomics & multi-omics analysis of Crohn’s disease. Gut.

[93] Xia J., Psychogios N., Young N., Wishart D.S. (2009). MetaboAnalyst: A web server for metabolomic data analysis and interpretation. Nucleic Acids Res..

[94] Ernest B., Gooding J.R., Campagna S.R., Saxton A.M., Voy B.H. (2012). MetabR: An R script for linear model analysis of quantitative metabolomic data. BMC Res. Notes.

[95] Alkhalifah Y., Phillips I., Soltoggio A., Darnley K., Nailon W.H., McLaren D., Eddleston M., Thomas C.L.P., Salman D. (2019). VOCCluster: Untargeted metabolomics feature clustering approach for clinical breath gas chromatography/mass spectrometry data. Anal. Chem..

[96] Rusilowicz M.J., Dickinson M., Charlton A.J., O’Keefe S., Wilson J. (2018). MetaboClust: Using interactive time-series cluster analysis to relate metabolomic data with perturbed pathways. PLoS ONE.

[97] Chanana S., Thomas C.S., Zhang F., Rajski S.R., Bugni T.S. (2020). HCAPCA: Automated hierarchical clustering and principal component analysis of large metabolomic datasets in R. Metabolites.

[98] Rogers A., McGeachie M., Baron R.M., Gazourian L., Haspel J.A., Nakahira K., Fredenburgh L.E., Hunninghake G.M., Raby B.A., Matthay M.A. (2014). Metabolomic derangements are associated with mortality in critically ill adult patients. PLoS ONE.

[99] Kelly R.S., McGeachie M.J., Lee-Sarwar K.A., Kachroo P., Chu S., Virkud Y.V., Huang M., Litonjua A.A., Weiss S.T., Lasky-Su J. (2018). Partial least squares discriminant analysis and Bayesian networks for metabolomic prediction of childhood asthma. Metabolites.

[100] Min S., Lee B., Yoon S. (2016). Deep learning in bioinformatics. Brief. Bioinform..

[101] Brereton R.G., Lloyd G.R. (2010). Support vector machines for classification and regression. Analyst.

[102] Touw W.G., Bayjanov J.R., Overmars L., Backus L., Boekhorst J., Wels M., van Hijum S.A. (2013). Data mining in the life sciences with random forest: A walk in the park or lost in the jungle?. Brief. Bioinform..

[103] Liebal U.W., Phan A.N.T., Sudhakar M., Raman K., Blank L.M. (2020). Machine learning applications for mass spectrometry-based metabolomics. Metabolites.

[104] Lee M.Y., Hu T. (2019). Computational methods for the discovery of metabolic markers of complex traits. Metabolites.

[105] Ghaffari M.H., Jahanbekam A., Sadri H., Schuh K., Dusel G., Prehn C., Adamski J., Koch C., Sauerwein H. (2019). Metabolomics meets machine learning: Longitudinal metabolite profiling in serum of normal versus overconditioned cows and pathway analysis. J. Dairy Sci..

[106] Zhang B., Horvath S. (2005). A general framework for weighted gene co-expression network analysis. Stat. Appl. Genet. Mol. Biol..

[107] Langfelder P., Horvath S. (2008). WGCNA: An R package for weighted correlation network analysis. BMC Bioinform..

[108] Zhu Y., Pei G., Niu X., Shi M., Zhang M., Chen L., Zhang W. (2015). Metabolomic analysis reveals functional overlapping of three signal transduction proteins in regulating ethanol tolerance in cyanobacterium *Synechocystis* sp. PCC 6803. Mol. BioSyst..

[109] Pei G., Chen L., Zhang W. (2017). WGCNA application to proteomic and metabolomic data analysis. Methods in Enzymology.

[110] Ritchie M.E., Phipson B., Wu D., Hu Y., Law C.W., Shi W., Smyth G.K. (2015). Limma powers differential expression analyses for RNA-sequencing and microarray studies. Nucleic Acids Res..

[111] Xia J., Mandal R., Sinelnikov I.V., Broadhurst D., Wishart D.S. (2012). MetaboAnalyst 2.0—A comprehensive server for metabolomic data analysis. Nucleic Acids Res..

[112] Xia J., Wishart D.S. (2016). Using MetaboAnalyst 3.0 for comprehensive metabolomics data analysis. Curr. Protoc. Bioinform..

[113] Chong J., Xia J. (2018). MetaboAnalystR: An R package for flexible and reproducible analysis of metabolomics data. Bioinformatics.

[114] Friedman J., Hastie T., Tibshirani R. (2010). Regularization paths for generalized linear models via coordinate descent. J. Stat. Softw..

[115] Gaude E., Chignola F., Spiliotopoulos D., Spitaleri A., Ghitti M., Garcia-Manteiga J.M., Mari S., Musco G. (2013). Muma, an R package for metabolomics univariate and multivariate statistical analysis. Curr. Metab..

[116] Uppal K., Soltow Q.A., Promislow D.E.L., Wachtman L.M., Quyyumi A.A., Jones D.P. (2015). MetabNet: An R package for metabolic association analysis of high-resolution metabolomics data. Front. Bioeng. Biotechnol..

[117] Mock A., Warta R., Dettling S., Brors B., Jäger D., Herold-Mende C. (2018). MetaboDiff: An R package for differential metabolomic analysis. Bioinformatics.

[118] De Livera A.M., Olshansky G., Simpson J.A., Creek D.J. (2018). NormalizeMets: Assessing, selecting and implementing statistical methods for normalizing metabolomics data. Metabolomics.

[119] Wolthuis J.C., Magnusdottir S., Pras-Raves M., Moshiri M., Jans J.J.M., Burgering B., van Mil S., de Ridder J. (2020). MetaboShiny: Interactive analysis and metabolite annotation of mass spectrometry-based metabolomics data. Metabolomics.

[120] Choudhary K.S., Fahy E., Coakley K., Sud M., Maurya M.R., Subramaniam S. (2020). MetENP/MetENPWeb: An R package and web application for metabolomics enrichment and pathway analysis in metabolomics workbench. bioRxiv.

