# Supplementary material for: Overview of Metabolomic Analysis and the Integration with Multi-Omics for Economic Traits in Cattle
Source: Metabolites. 2021 Oct 30;11(11):753. doi: 10.3390/metabo11110753 (PMC8621036; doi:10.3390/metabo11110753)
Supplement: Supplementary file 1 [file metabolites-11-00753-s001.zip › metabolites-1421626-supplementary.pdf]

Supplementary Table S1. Significantly metabolic pathways (FDR < 0.05) for the metabolites associated with feed efficiency using *Bos taurus* as the library.

Pathway name	Matched metabolite ratio	Metabolite	*P*-value	-Log(*P*-value)	False discovery rate (FDR)	Pathway impact value
Aminoacyl-tRNA biosynthesis	6/48	Glutamate, Glycine, Lysine, Phenylalanine, Threonine, Tyrosine	3.78×10^{-5}	4.42	2.64×10^{-3}	0
Glyoxylate and dicarboxylate metabolism	5/32	Acetate, Citrate, Formate, Glutamate, Glycine	6.28×10^{-5}	4.20	2.64×10^{-3}	0.138
Phenylalanine metabolism	3/12	Hippurate, Phenylalanine, Tyrosine	5.41×10^{-4}	3.27	0.0115	0.357
Alanine, aspartate and glutamate metabolism	4/28	α-ketoglutarate, Citrate, Glutamate, Succinate	5.48×10^{-4}	3.26	0.0115	0.245
Butanoate metabolism	3/15	α-ketoglutarate, Glutamate, Succinate	1.09×10^{-3}	2.96	0.0143	0
Glycine, serine and threonine metabolism	4/34	Betaine, Creatine, Glycine, Threonine	1.17×10^{-3}	2.93	0.0143	0.342
Phenylalanine, tyrosine and tryptophan biosynthesis	2/4	Phenylalanine, Tyrosine	1.19×10^{-3}	2.92	0.0143	1.0
D-Glutamine and D-glutamate metabolism	2/5	α-ketoglutarate, Glutamate	1.97×10^{-3}	2.71	0.0207	1.0
Citrate cycle (TCA cycle)	3/20	α-ketoglutarate, Citrate, Succinate	2.60×10^{-3}	2.59	0.0243	0.182

Note: The pathway impact value is calculated using the sum of importance measures of the matched metabolites divided by the sum of the importance measures of all metabolites.

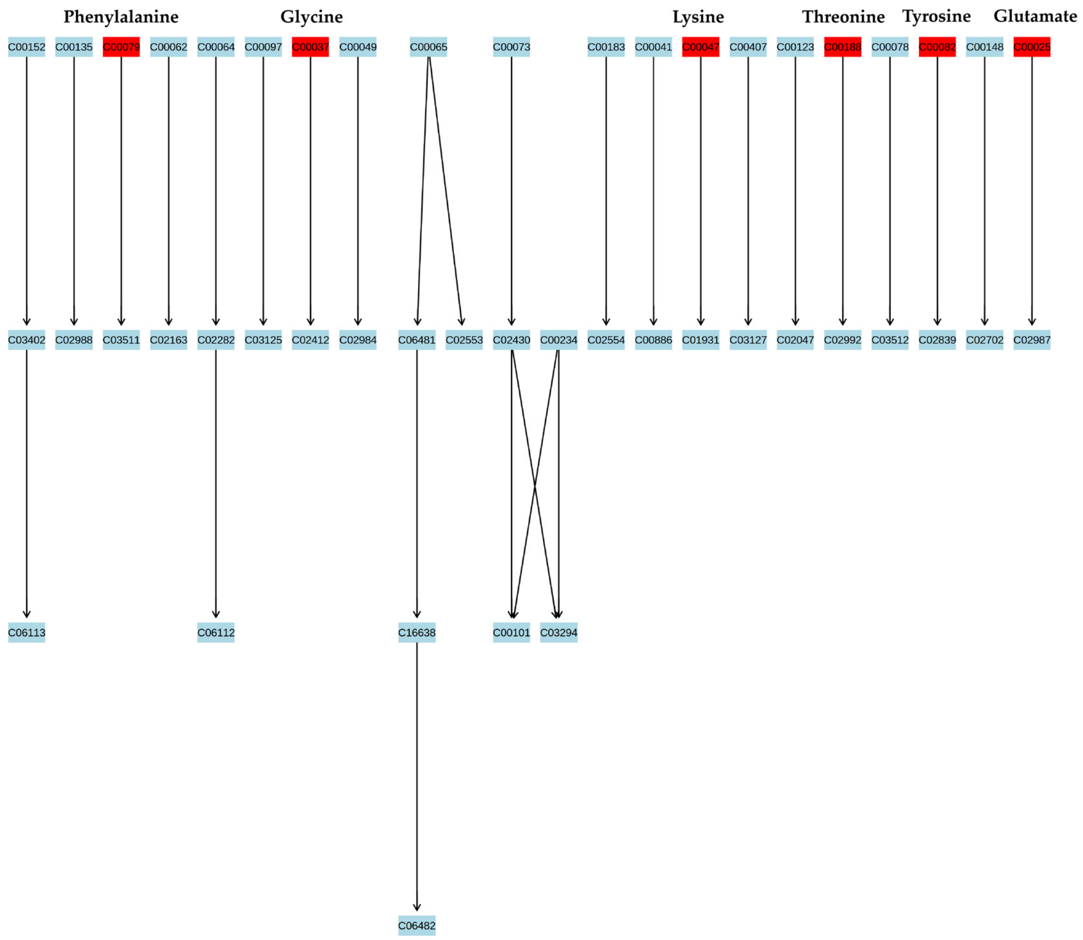

Supplementary Figure S1. The connections of the metabolites including glutamate, glycine, lysine, phenylalanine, threonine and tyrosine (red color) enriched in the aminoacyl-tRNA biosynthesis pathway.
